# Supplementary material for: Glia Cells Are Selectively Sensitive to Nanosized Titanium Dioxide Mineral Forms
Source: Int J Mol Sci. 2025 Oct 4;26(19):9684. doi: 10.3390/ijms26199684 (PMC12524891; doi:10.3390/ijms26199684)
Supplement: Supplementary file 1 [file ijms-26-09684-s001.zip › Supplementary Materials.pdf]

Glia Cells Are Selectively Sensitive to Nanosized Titanium Dioxide Mineral Forms

Supplementary Figure S1

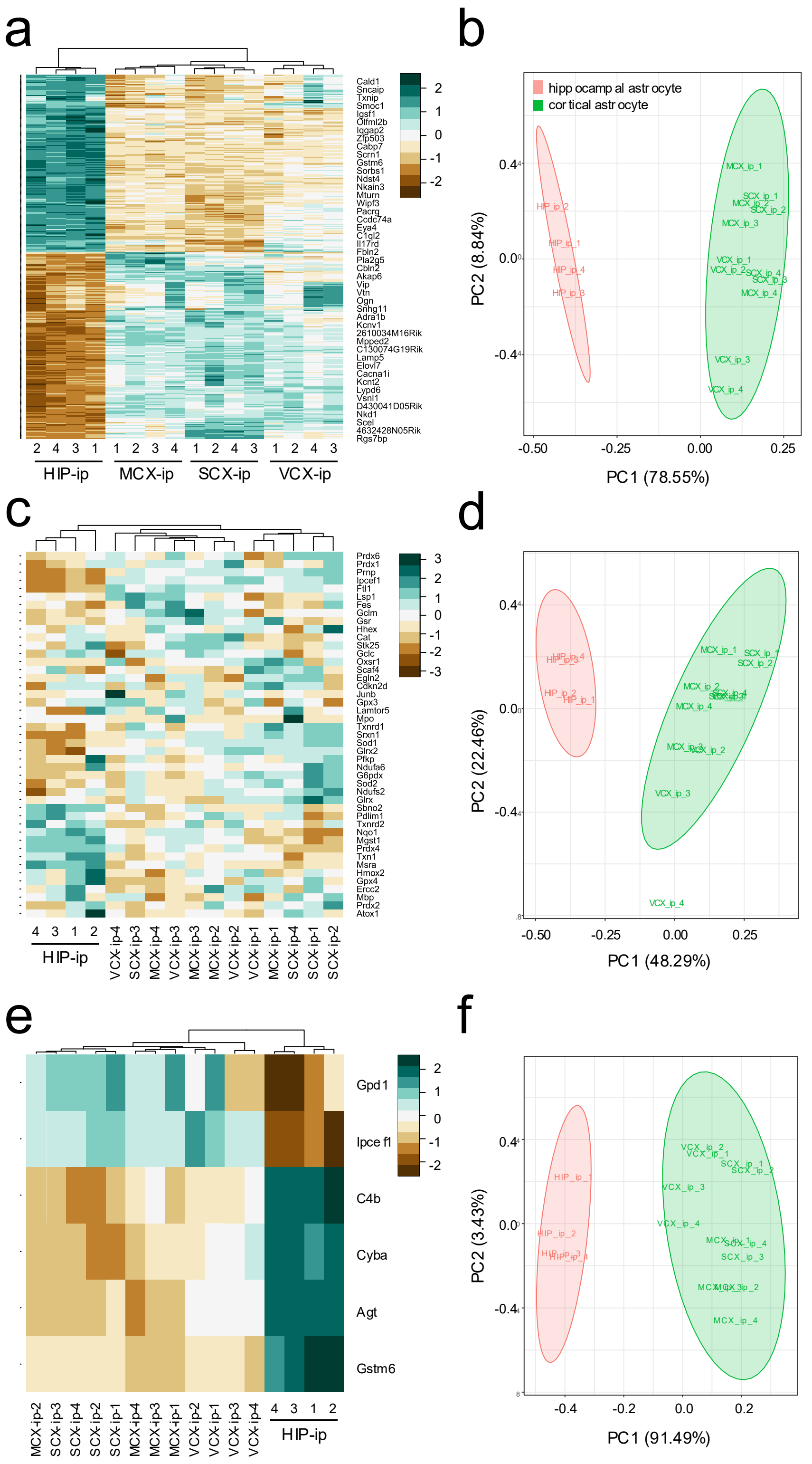

Supplementary Figure S1. Hippocampal and cortical astrocytes differ in their general and oxidative stress transcriptomic profile. (**a**) Heat map showing the log_2 fold change (FC) values of 359 differentially expressed genes (DEGs) between hippocampal (HIP) and cortical (from motor, sensory, and visual cortex - MCX, SCX and VCX, respectively) origins, with a threshold of log2 FC > 1, FDR < 0.05. "ip-#" represent the biological replicates in the original GSE198024 dataset [1]. According to the dendrogram, astrocytes can be classified into 2 broad groups which is confirmed by the (**b**) PCA analysis. (**c**) Heat map showing the log_2 expression values of 45 expressed genes from the Hallmark Reactive Oxygen Species Pathway GSEA gene set (MM3895, GSEA) in the same hippocampal (HIP) and cortical (MCX, SCX and VCX) samples. The dendrogram again shows that astrocytes cluster into two broad groups, which is confirmed by the (**d**) PCA analysis. (**e**) Heat map showing the log_2 fold change (FC) values of six differentially expressed genes (DEGs) between hippocampal (HIP) and cortical (MCX, SCX and VCX) samples filtered using the DEGS with Oxidative Stress and redox pathway-related gene sets (see Table A1) with a threshold of log2 FC > 1, FDR < 0.05. The dendrogram shows the classification of astrocytes could be classified into 2 broad groups, confirmed by the (**f**) PCA analysis.

Reference

1. Endo, F.; Kasai, A.; Soto, J.S.; Yu, X.; Qu, Z.; Hashimoto, H.; Gradinaru, V.; Kawaguchi, R.; Khakh, B.S. Molecular Basis of Astrocyte Diversity and Morphology across the CNS in Health and Disease. *Science* 2022, *378*, eadc9020, doi:10.1126/science.adc9020.

Supplementary File S1

Supplementary File S1_tabular.tsv: List of differentially expressed genes among hippocampal and cortical glia samples originated from the GSE198024 bulk RNA-seq, normalized expression dataset published by Endo et al (2022) [31]. The list generated using the edgeR package (v3.36), which included filtering of low-expression genes (CPM < 1), dispersion estimation, and exact test-based comparison. Genes with a false discovery rate (FDR) < 0.05 and absolute log_2 fold change (|log2FC|) > 1 were considered significantly differentially expressed.

Supplementary Table S1

List of differentially expressed genes among hippocampal and cortical glia samples related to oxidative stress and glutathione metabolism related gene sets from GSEA Molecular Signature Database. Gene set of differentially expressed genes (Supplementary File 1) was systematically filtered with oxidative stress and glutathione metabolism related gene sets from GSEA Molecular Signature Database (Appendix Table A1).

Table S1. List of differentially expressed genes among hippocampal and cortical glia samples related to with oxidative stress and glutathione metabolism related gene sets from GSEA Molecular Signature Database.

GeneID	logFC	logCPM	F	PValue	FDR
Agt	2.913289	6.493155	866.4965	9.30E-17	3.07E-13
Gstm6	1.055324	3.754909	175.0624	3.37E-11	5.58E-09
C4b	2.032913	6.945142	225.4295	1.01E-10	1.31E-08
Cyba	1.612764	2.814263	150.6637	1.25E-10	1.55E-08
Ipcef1	-1.51417	3.381105	129.7086	4.56E-10	4.30E-08
Gpd1	-1.13196	6.938677	32.37137	4.99E-05	0.000516
